# Trade-offs in proton and photon radiotherapy for pituitary adenomas

**DOI:** 10.1016/j.phro.2026.100994

**Published:** 2026-05-10

**Authors:** David J.A. Palm, John Paulissen, Rik Emmah, David Hofstede, Nikolina E. Birimac, Ruud Houben, Marlies E. Granzier, Marloes Nogarede, Ans C.C. Swinnen, Jasper van Aalst, Yasin Temel, Marleen Kars, Daniëlle B.P. Eekers, Catharina M.L. Zegers, Inge Compter

**Affiliations:** a Department of Radiation Oncology (Maastro), GROW Research Institute for Oncology and Reproduction, Maastricht University Medical Centre+, Maastricht, the Netherlands; b Department of Neurosurgery, Maastricht University Medical Center+, Maastricht, the Netherlands; c Department of Internal Medicine, Division of Endocrinology, Maastricht University Medical Center+, Maastricht, the Netherlands

**Keywords:** Radiotherapy, Photons, Protons, Coplanar, Non-coplanar, Pituitary

## Abstract

**Background and Purpose:**

Pituitary Adenomas (PAs) are tumours proximal to sensitive organs of interest (OOIs). These tumours may be well-suited for proton therapy due to the Bragg peak. Few studies have evaluated the potential dose-volume benefits of protons for PAs in the pencil beam scanning era.

**Materials and Methods:**

Treatment plans for 15 PA patients were made using intensity modulated proton therapy (IMPT), coplanar- and- non-coplanar photon therapy. Dose distributions to OOIs were compared using the Radiation Oncology Collaborative Comparison Group’s (ROCOCO) Performance Scoring System (RPSS), which estimates adverse event (AE) burden.

**Results:**

Proton therapy resulted in significantly lower overall estimated AE burden than both photon modalities (median RPSS: 1.09 vs. 1.24; p < 0.005). This reduction was due to significantly lower D_40%_ to the hippocampi. For the right hippocampus, median D_40%_ (EQD_Gy2_) was 1.1 Gy with IMPT, compared with 4.9 Gy for coplanar and 3.2 Gy for non-coplanar photons. Doses with protons and non-coplanar photons were significantly lower than those for coplanar photons (p < 0.001). For the left hippocampus, IMPT achieved a median D_40%_ of 0.7 Gy, compared with 5.0 Gy for coplanar and 2.7 Gy for non-coplanar photons. Non-coplanar was significantly lower than coplanar (p < 0.001), while IMPT was significantly lower than both photon modalities (p < 0.001). Protons delivered higher doses to the brainstem, optic nerves, chiasm, and the hypothalami.

**Conclusions:**

These findings indicate that IMPT can significantly reduce AEs to critical brain structures. Although considerations of cost, accessibility, and clinical validation remain, IMPT may offer a meaningful therapeutic advantage.

## Highlights

• Protons reduced hippocampal dose by 3–7-fold compared to photons.
• Protons (1.09) had lower estimated adverse events compared to photons (1.24).
• Optic nerve dose was increased ≥ 65 % with protons compared to photon techniques.

## Graphical abstract

**Figure:**
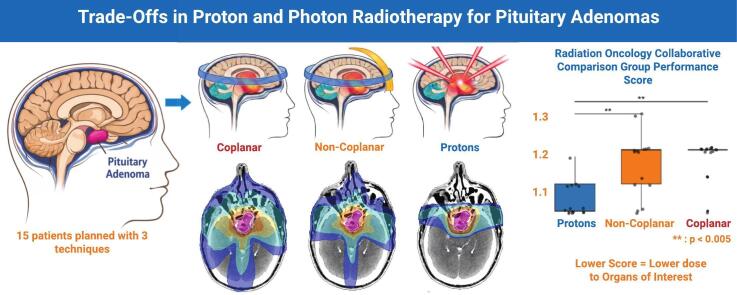

## 1 Introduction

Pituitary adenomas (PAs) are the most frequently occurring tumours of the sellar region, present in 10–22 % of the general population [1], [2]. These tumours often remain undiagnosed, as less than 1 % will require medical intervention [1], [2], [3]. First-line therapy for symptomatic PAs is transsphenoidal resection. Prolactinomas are an exception, typically managed with dopamine agonists [1], [4]. Second- or third-line therapy may include radiotherapy (RT), delivered as a single fraction or via a hyperfractionated approach [5], [6], [7], [8], [9], [10], [11]. Stereotactic radiosurgery (SRS) is suitable for PAs < 2.5 cm and safely distant from the optic chiasm [10], [12], [13], [14]. For larger PAs near the optic apparatus, hyperfractionated RT is preferred to minimize the risk of optic damage, particularly radiation-induced optic neuropathy (RION) [13], [15], [16], [17].

Pituitary adenomas are located deep in the brain, near the skull-base and several critical organs of interest (OOIs). This proximity makes it challenging to administer the therapeutic dose to the tumour while sparing OOIs from RT-induced adverse events (AEs). Multiple RT approaches can be used for PA management: coplanar photon fractionated stereotactic radiotherapy (FSRT) with volumetric arc therapy (VMAT), non-coplanar FSRT VMAT, intensity modulated proton therapy (IMPT), and carbon ion therapy. Non-coplanar FSRT enables beam delivery from multiple planes by rotating the treatment couch, reducing beam overlap compared to coplanar FSRT [18]. This approach can improve dose conformity and decrease exposure to OOIs, potentially lowering AEs. Proton therapy is increasingly used to reduce dose to healthy tissues for various neuro-oncological indications such as gliomas, meningiomas, and skull base tumours [19]. Proton therapy has been used to treat PAs since the 1950s [20], [21], [22], [23], [24]. The Bragg Peak allows protons to deposit most of their energy at a specific depth, reducing radiation exposure to surrounding tissues [25], [26], [27]. Despite its dose-volume advantages and proven efficacy equivalence to photon RT, the clinical benefit of proton RT over photon-based RT is an active area of research [28].

Few studies have investigated the potential effects of these modality-specific dose differences in the modern era, with very limited work addressing PAs. Investigations are limited by their cohort size, mixing with other tumour groups, or focus on radiosurgically treated patients [29], [30], [31]. Therefore, the aim of this study was to evaluate whether the expected dose-volume advantages of proton therapy over photon RT translate into clinically meaningful reductions in AEs in fractionated RT in the modern era of radiotherapy. To this end, an in-silico treatment planning investigation was conducted to compare the estimated AE burden of VMAT verses IMPT in the treatment of PAs based on the Radiation Oncology Collaborative Comparison Group (ROCOCO) Performance Scoring System (RPSS).

## 2 Materials and methods

### 2.1 Population

The cohort consisted of 15 PA patients who were treated with fractionated RT at MAASTRO, Maastricht, the Netherlands, between 2017 and 2021. For each patient, one IMPT plan and two photon FSRT plans, one coplanar and one non-coplanar, were constructed. The main inclusion criteria was the availability of a planning CT without metal or contrast artefacts to enable both photon and proton treatment planning. An overview of patient and treatment characteristics is provided in Table 1. This in silico study received approval from the local institutional review board (research number P0524, date November 2nd, 2021). The local ethics committee waived the requirement for informed consent in accordance with Dutch legislation, because this retrospective in silico study was based solely on fully anonymized data, involved no direct patient contact, and entailed minimal risk for participants.

**Table 1:** Overview of patient characteristics. Age and gross tumour volume (GTV) denoted in median, range in brackets.

Subcategory	N
**PA**	
− Secretory	5
− Non-Secretory	10
**Age**	**Median (range)**
Diagnosis	57 (45–74)
Start RT	64 (51–85)
**Prior Management**	
1 Resection	8
2 Resections	2
Medical	2
None	2
Radiotherapy & Resection	1
**RT Treatment**	
− 28 Fractions	10
− 29 Fractions	5
**Tumour Size**	**Median (Range)**
GTV (cm^3^)	8.69 (0.64 – 21.35)

### 2.2 Target volume definition and delineation

Prior to RT, each patient underwent a planning computed tomography (CT) scan with 1 mm slices (Siemens SOMATOM Confidence, Siemens Healthcare, Erlangen, Germany) with contrast while immobilized using a double-shell thermoplastic head mask (Macromedics Holding B.V., Moordrecht, The Netherlands) and knee fix. Additionally, a Gadolinium-enhanced T1-weighted magnetic resonance image (MRI) (Ingenia CX / Achieva MR systems, Philips Healthcare, Eindhoven, The Netherlands) was acquired. The gross tumour volume (GTV) was delineated by an experienced neuro radiation oncologist based on the fused Gadolinium-enhanced MRI and contrast-enhanced CT according to the European Society for Radiotherapy and Oncology Advisory Committee in Radiation Oncology Practice (ESTRO ACROP) guidelines and was confirmed by a second experienced radiation oncologist [32]. No margin expansion was applied for the clinical target volume (CTV), so the CTV was equal to the GTV. A 1 mm planning target volume (PTV) margin was added to account for setup uncertainties for FSRT in our centre. The OOIs were delineated by a RT technician according to the European Particle Therapy Network (EPTN) neuro contouring atlas and was confirmed by a radiation oncologist [33].

### 2.3 Dose prescription

The prescribed dose to the target volume was either 50.4 Gy or 52.2 Gy,^1^ depending on the tumour type. Non-secretory tumours were treated in 28 fractions, and secretory tumours in 29 fractions, each with 1.8 Gy per fraction. A factor of 1.1 was applied to account for the increased relative biological effectiveness (RBE) of IMPT. All treatment plans were designed to ensure that the delineated OOIs never exceeded the dose guidance according to the EPTN guidelines [34]. To ensure each technique was utilized to its full potential, plans were generated by expert planners specializing in each specific modality. During RT planning, the as low as reasonably achievable principle (ALARA) was strictly followed. In addition, optimization dose guidance was standardized to avoid planner bias.

### 2.4 Photon and proton treatment plans

**Fig. 1:**
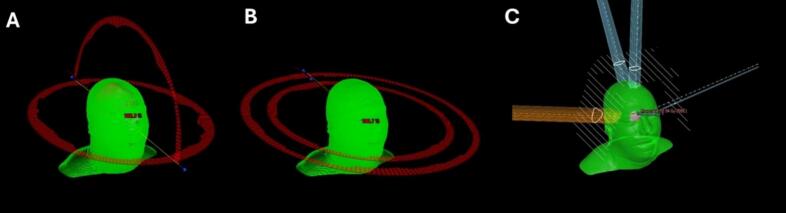
Beam configurations for different radiotherapy plans. Panel A: Non-coplanar plan with two beams — one whole and one half-arc. Panel B: Coplanar plan with two whole-arc beams. Panel C: Proton plan configuration with four beams.

The co- and non-coplanar FSRT plans were created using Eclipse version 15 (Varian Medical Systems, Palo Alto, CA) with VMAT and Acuros XB dose calculation algorithm (version 15.5.11). A maximum of 4 beams as whole or half-arcs were used for non-coplanar FSRT, while at most 2 whole arcs were used for coplanar FSRT. Each FSRT plan was normalized to ensure that at least 99 % of the PTV received 95 % of the dose. The co- and non-coplanar beam arrangements for a typical patient are shown in Fig. 1.

Proton therapy plans were created using RayStation (Version 2024B, RaySearch Laboratories, Stockholm, Sweden) using IMPT with pencil beam scanning (PBS). A setup uncertainty margin of 1 mm was applied for the CTV, with 3 % range uncertainty. It was ensured that D_98%_ ≥ 95 % for all plans. Furthermore, plans were created to be robustly optimized with voxel-wise minimum and maximum evaluation; 21 scenarios were evaluated per patient [35]. A minimum of three beams were used to construct the proton plans. The clinical Monte Carlo engine V5.7 was used in the treatment planning system.

The radiation beams were arranged individually to achieve the lowest dose possible to the OOIs and avoid passing through air cavities. At least 98 % of the CTV received 94 % of the prescribed dose in the worst-case distribution (i.e., the voxel-wise minimum across all uncertainty scenarios). For robust planning, it was ensured that 98 % of the CTV received at least 95 % of the dose. The air gap between the nozzle snout and the patient surface was fixed at 5 cm for all fields. The minimum energy of the beam model was 45 MeV with a spot size sigma ranging from 1.8 mm at 45 MeV to 16 mm at 227 MeV [36].

### 2.5 The ROCOCO performance scoring system

In this investigation, the RPSS was applied to quantify and compare the estimated AE burden between photon FSRT and IMPT [18]. The RPSS is a dimensionless ratio; a higher RPSS represents a higher risk of RT-induced AEs. The pituitary gland was excluded from the RPSS in this study, as it contained the primary target volume. Before calculating the RPSS, the dose to the OOIs extracted from the treatment planning systems were recalculated into equivalent dose in 2-Gray fractions (EQD_Gy2_) with the linear quadratic formula [37]. The RPSS was calculated per patient for each modality. One patient underwent bilateral cataract surgery prior to RT; hence the lenses are excluded in the RPSS score. The dose to OOIs which are not included in the RPSS were also extracted and investigated. These OOIs are not included in the RPSS due to the expectation that their dose guidance are never surpassed in the clinic.

### 2.6 Statistical analysis

Statistical analysis was performed in R (version 4.3.1) using the tidyverse and ggplot2 packages [38]. The RPSS of the coplanar, non-coplanar and proton RT modalities were compared using the Friedman test. In case of overall significance, results were further investigated with pairwise Wilcoxon tests with Bonferroni correction. A p-value < 0.05 was considered significant. Additionally, it was also investigated whether the different RT planning approaches resulted in significant differences in dose to the whole brain, surface and interior of the brainstem, optic nerves, chiasm, retinae, vestibulocochlear complexes, and the anterior cerebellum. Finally, the integral dose is also reported and was defined as the mean dose to the body contour minus the GTV.

## 3 Results

Data from 45 treatment plans of 15 patients across three modalities were analysed.

**Fig. 2:**
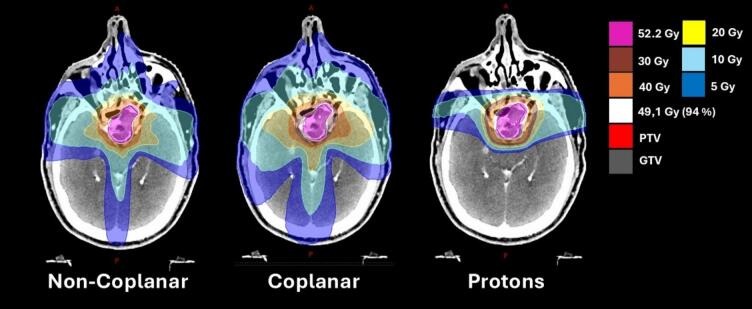
Dose colour-wash images with isodose lines overlaid on the planning CT scan. Dose levels are indicated in the top right. The GTV, PTV, and D_94%_ are also visualized.

Examples of the RT dose colour wash is highlighted in Fig. 2.

**Fig. 3:**
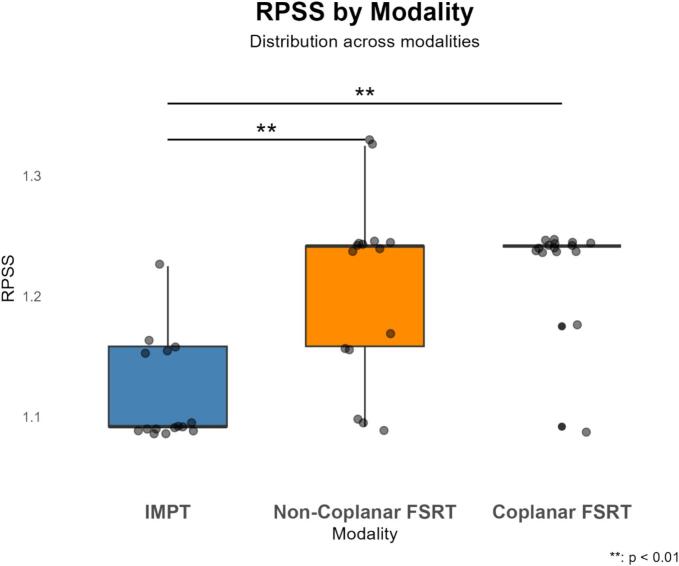
RPSS distribution by modality. Box-and-whisker plots representing the median and the 25th and 75th percentiles. Individual patient data are overlaid as grey jittered dots. Median (interquartile range (IQR)) values: IMPT, 1.09 (1.09–1.16); Non-coplanar FSRT, 1.24 (1.16–1.24); and Coplanar FSRT, 1.24 (1.24–1.24).

With respect to the RPSS, IMPT was the superior modality overall. IMPT led to the lowest RPSS in twelve cases. In two cases (#7 and #10), IMPT was equivalent to non-coplanar FSRT but was better than coplanar FSRT. In the final case (#15), all modalities scored equally. The overall RPSS results are visualized in Fig. 3, while the individual results are visualized in Supplementary Fig. S1.

The Friedman test revealed a significant overall difference in RPSS scores between modalities. Pairwise comparisons using the Wilcoxon signed-rank test showed that IMPT led to a significant (p < 0.001) reduction in RPSS compared to both coplanar and non-coplanar FSRT plans (p < 0.001). No significant difference was observed between coplanar non-coplanar FSRT (p = 1.00), see Fig. 3.

**Fig. 4:**
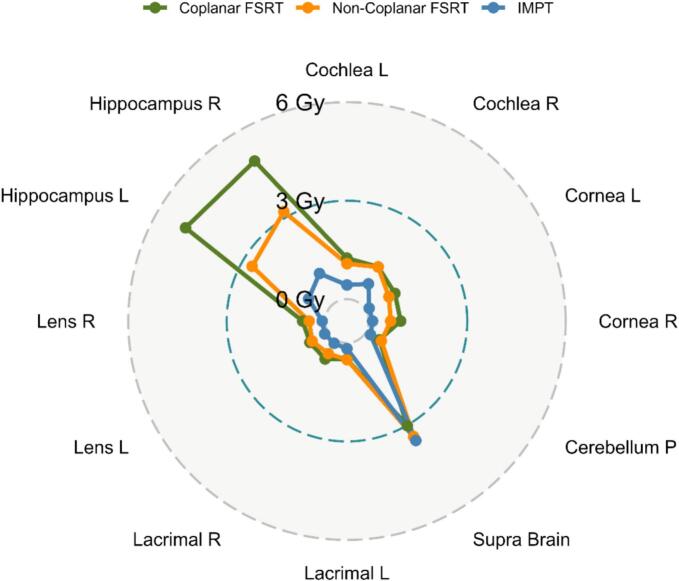
Median EQD_Gy2_ to the OOI included in the RPSS model. L: Left, R: Right, P: Posterior, Supra: Supratentorial.

The D_40%_ to the hippocampi was the main factor influencing the RPSS in this cohort. The median hippocampal doses (D_40%_) with IMPT were 0.7 Gy (range 0.2–2.8) for the left and 1.0 Gy (0.1–8.3) for the right. For non-coplanar FSRT, median doses were 2.7 Gy (1.8–5.4) and 3.2 Gy (1.3-6.3) for the left and right, respectively. Coplanar FSRT delivered significantly higher doses: 5.0 Gy (1.8–7.3) for the left and 4.9 Gy (1.7–8.7) for the right when compared to non-coplanar and IMPT (p < 0.001). Furthermore, IMPT dose was significantly lower than non-coplanar FSRT for the left hippocampus (p < 0.001), but not the right (p > 0.1). Finally, a paired two-tailed *t*-test showed no significant difference between left and right hippocampal D_mean_ and D_40%_ within a modality (all p > 0.05). Fig. 4 summarizes the median doses to the OOIs included in the RPSS.

Notable variations were observed in dose to OOIs which are not included in the RPSS. Friedman testing revealed that there was a significant overall difference in every OOI tested except for the whole brain. Post-hoc Wilcoxon testing revealed that protons delivered significantly higher dose than both photon modalities to the chiasm, optic nerves, hypothalami, and the surface of the brainstem. Furthermore, protons significantly spared the left and right VSCC, and the anterior cerebellum compared to both photon modalities. An overview of the EQD_Gy2_ values per modality is provided in Table 2. The result of each post-hoc test is provided in Supplementary Table S1.

**Table 2:** Median dose (EQD_Gy2_) per OOI, with Friedman test results assessing overall significant differences. *: Significant overall difference.

**OOI**	**IMPT** Median (IQR) EQD_Gy2_	**Non-coplanar** Median (IQR) EQD_Gy2_	**Coplanar** Median (IQR) EQD_Gy2_	**Friedman**
Brain	47.8 (5.3)	46.9 (1.8)	47.2 (1.4)	2.82e-01
Brainstem interior	37.4 (17.3)	28.7 (16.3)	36.2 (13.9)	3.04e-05*
Brainstem surface	42.7 (13.6)	34.1 (20.6)	39.1 (15.1)	3.06e-07*
Chiasm	49.9 (2.2)	45.5 (4.6)	45.7 (5.9)	1.12e-05*
Optic nerve left	41.2 (7.8)	21.9 (18.9)	24.3 (20.6)	8.57e-06*
Optic nerve right	42.1 (11.8)	24.5 (25.8)	25.1 (23.5)	1.41e-04*
Hypothalamus left	21.8 (17.4)	18.0 (11.6)	11.6 (8.8)	2.57e-04*
Hypothalamus right	22.8 (15.4)	14.9 (12.6)	11.6 (11.3)	6.77e-05*
Vestibulocochlear complex left	0.1 (0.3)	0.9 (0.3)	1.2 (0.6)	3.37e-06*
Vestibulocochlear complex right	0.2 (0.2)	1.0 (0.3)	1.2 (0.6)	1.55e-03*
Retina left	0.5 (1.6)	2.1 (1.2)	2.9 (2.7)	2.32e-03*
Retina right	0.7 (2.6)	1.9 (1.6)	3.5 (2.6)	5.72e-04*
Cerebellum anterior	0.8 (1.0)	4.5 (4.0)	7.9 (5.4)	2.85e-05*

Box-and-whisker plots illustrating the dose distributions for these OOIs (except the VSCC) are shown in Supplementary Figs. S2-S5. Additionally, the cumulative dose-volume histograms (DVH) of the optical system and the hippocampi for a representative patient are presented Supplementary Figs. S6 and S7.

Finally, proton plans exhibited the highest integral dose, with a median of 1.9 Gy, which was significantly higher than both the coplanar (1.3 Gy, p < 0.001) and non-coplanar (1.6 Gy, p < 0.001) FSRT plans. Furthermore, the non-coplanar plans demonstrated a significantly higher integral dose compared to coplanar FSRT (p < 0.0001), while protons resulted in significantly higher integral dose than both photon modalities (p < 0.0001). The results of the integral dose for each treatment modality are presented in Supplementary Fig. S8.

## 4 Discussion

This in-silico treatment planning study investigated the estimated AE burden between photon-versus proton-based RT in the management of PAs. Protons led to a significant decrease in the RPSS compared to coplanar and non-coplanar FSRT. Conversely, both photon RT techniques delivered significantly lower doses to the chiasm, optic nerves, and the surface of the brainstem compared to IMPT.

Previous literature comparing photon and proton RT in PA has shown varied results. Bolsi et al., performed an in-silico investigation on 12 patients with acoustic neurinomas, meningiomas and PAs [29]. Their findings showed that proton RT was superior to photon RT with respect to target coverage, uniformity, and OOIs sparing. Proton radiotherapy led to significant decreases in dose to the chiasm and optic nerves, opposite to our findings. Another investigation re-planned a single PA case using eight different techniques and found that conformal proton therapy was the preferred RT technique due to a lower integral dose, indicating a reduced risk of second tumour formation [30]. In a recently published investigation, Heggebø et al., conducted a study similar to the one presented here. They performed an in-silico analysis comparing dose-volume differences between VMAT and 2-and 3-field IMPT for PA management [39]. Similar to our findings, they reported that IMPT significantly reduced the D_40%_ to the hippocampi. In contrast, they reported higher doses to the cornea, retinae, lenses and lacrimal glands with VMAT. Additionally, significant differences in dose to the chiasm and the optic nerves were observed here, while the previous publication did not. Finally, our results indicate that protons exhibited a higher integral dose, suggesting a potentially increased risk of secondary malignancies. However, Heggebø et al., reported the opposite trend. These discrepancies may be explained by methodological differences, particularly the 5 mm GTV-to-CTV expansion used in their study compared with the 1 mm planning margins of this study. The larger margin likely increased the overlap between high-dose region and the body contour, thereby reducing the integral dose. It should also be noted that the integral dose was very low across all modalities. Taken together, these findings underscore the influence of evolving radiotherapy techniques, planning parameters, and patient-specific factors on dose-volume outcomes in PAs.

This investigation builds on the current body of evidence by calculating RPSS scores in an attempt to translate dose to OOIs to estimated AE burden. Reduced D_40%_ to the hippocampi was the primary driver for variations in the RPSS score. In this investigation it was observed that hippocampal dose was not completely symmetrical despite a centrally located tumour. Asymmetries in hippocampal dose were correlated with tumour laterality, and the subsequent optimization of beam angles. Dose to the hippocampi is strongly correlated with cognitive and memory impairment [40], [41], [42], [43], [44], [45], [46]. Reducing dose to the hippocampi is paramount in PA patients for preserving cognition, as long-term survival is expected [47], [48]. However, some of the findings warrant careful consideration.

Proton RT resulted in significantly higher dose to the surface and interior of the brainstem, the hypothalami, and the optic apparatus. Although the dose guidance for these OOIs were never exceeded, this finding does raise some concern. In clinical IMPT planning, the RBE is fixed at 1.1 due to the inability to model the variable RBE at the distal end of the proton beam [49], [50]. Moreover, the use of a fixed RBE of 1.1 does not account for potential increases in biological effectiveness associated with elevated linear energy transfer (LET) near the distal edge of the Bragg peak, which may lead to higher effective doses in adjacent critical structures and could increase the risk of toxicities such as RION [51].

Few studies have investigated the relation between IMPT and RION. One investigation on RION occurrence in skull base tumours treated with protons found < 1 % incidence below 60 Gy_RBE_ [52]. Another study determined that high-dose IMPT is safe considering its low incidence (6 %) when delivering ≥ 45 Gy_RBE_ to the optic nerves [53]. In spite of its established safety, these studies demonstrate that RION can occur below the current established dose guidance of 55 Gy_RBE_ [54]. With limited understanding of the biological mechanisms underpinning this phenomenon, it is important to remain cautious when approaching these higher doses to the optic apparatus.

Another structure in the vicinity of the pituitary is the hypothalamus. Literature indicates a threshold dose of around 20 Gy to this brain structure may cause insufficiency, possibly leading to endocrine and sleep disorders, changes in eating behaviour and metabolic disorders [55], [56], [57], [58]. In this treatment planning study, coplanar FSRT delivered significant lower doses to the hypothalamus, compared to non-coplanar FSRT and IMPT. Importantly, the hypothalami were delineated after all treatment plans were constructed. Hence these structures were not taken into account for the ALARA principle during RT planning.

This study has limitations affecting interpretation and generalizability. Photon plans used 1 mm PTV margins, which may not be feasible in all centres. The RPSS estimates AEs from literature-based models, potentially overlooking interpatient variability, and relies on expert-assigned weights without prospective validation. Additionally, the analysis was based on a small cohort of 15 previously treated PA patients from a single institution, which may not reflect the full spectrum of tumour presentations or anatomical variations and introduces potential selection bias.

While all plans respected clinical dose guidance, our findings compared to previous investigations suggest that reducing GTV-PTV/CTV margins has a large impact on OOIs sparing. Achieving such smaller margins in clinical practice will require advanced imaging, 6D couch positioning, non-coplanar planning, and robust motion management. Therefore, future efforts should prioritize integrating margin-reduction strategies into clinical standards, alongside careful modality selection.

In summary, this in-silico study demonstrates that both IMPT and non-coplanar FSRT can significantly reduce hippocampal dose compared with coplanar FSRT, suggesting a potential benefit for cognitive function preservation in patients with favourable prognosis. To establish the true clinical relevance of these dose-volume advantages, prospective validation is essential. A future research pathway should include integration of neurocognitive and quality-of-life assessments, as well as longitudinal correlation of dose-volume parameters with patient outcomes.

## 5 Declaration of generative AI and AI-assisted technologies in the manuscript preparation process

During the preparation of this work the authors used ChatGPT, Claude AI & Gemini to review text & script R. After using this tool/service, the authors reviewed and edited the content as needed and take full responsibility for the content of the published article.

## CRediT authorship contribution statement

**David J.A. Palm:** Writing – review & editing, Writing – original draft, Visualization, Methodology, Investigation, Formal analysis, Data curation. **John Paulissen:** Writing – review & editing, Writing – original draft. **Rik Emmah:** Writing – review & editing, Software. **David Hofstede:** Writing – review & editing, Software. **Nikolina E. Birimac:** Writing – review & editing, Software. **Ruud Houben:** Writing – review & editing, Methodology. **Marlies E. Granzier:** Writing – review & editing, Software, Methodology, Investigation. **Marloes Nogarede:** Writing – review & editing, Methodology. **Ans C.C. Swinnen:** Writing – review & editing. **Jasper van Aalst:** Writing – review & editing. **Yasin Temel:** Writing – review & editing. **Marleen Kars:** Writing – review & editing. **Daniëlle B.P. Eekers:** Writing – review & editing, Writing – original draft, Supervision. **Catharina M.L. Zegers:** Writing – review & editing, Writing – original draft, Validation, Supervision. **Inge Compter:** Writing – review & editing, Writing – original draft, Supervision, Methodology, Conceptualization.

## Declaration of competing interest

The authors declare that they have no known competing financial interests or personal relationships that could have appeared to influence the work reported in this paper.

## Footnotes

1 Throughout this paper, Gy_RBE_ reflects the ICRU93 definition.
