# Supplementary material for: Trade-offs in proton and photon radiotherapy for pituitary adenomas
Source: Phys Imaging Radiat Oncol. 2026 May 10;39:100994. doi: 10.1016/j.phro.2026.100994 (PMC13200076; doi:10.1016/j.phro.2026.100994)
Supplement: Supplementary Data 1 [file mmc1.pdf]

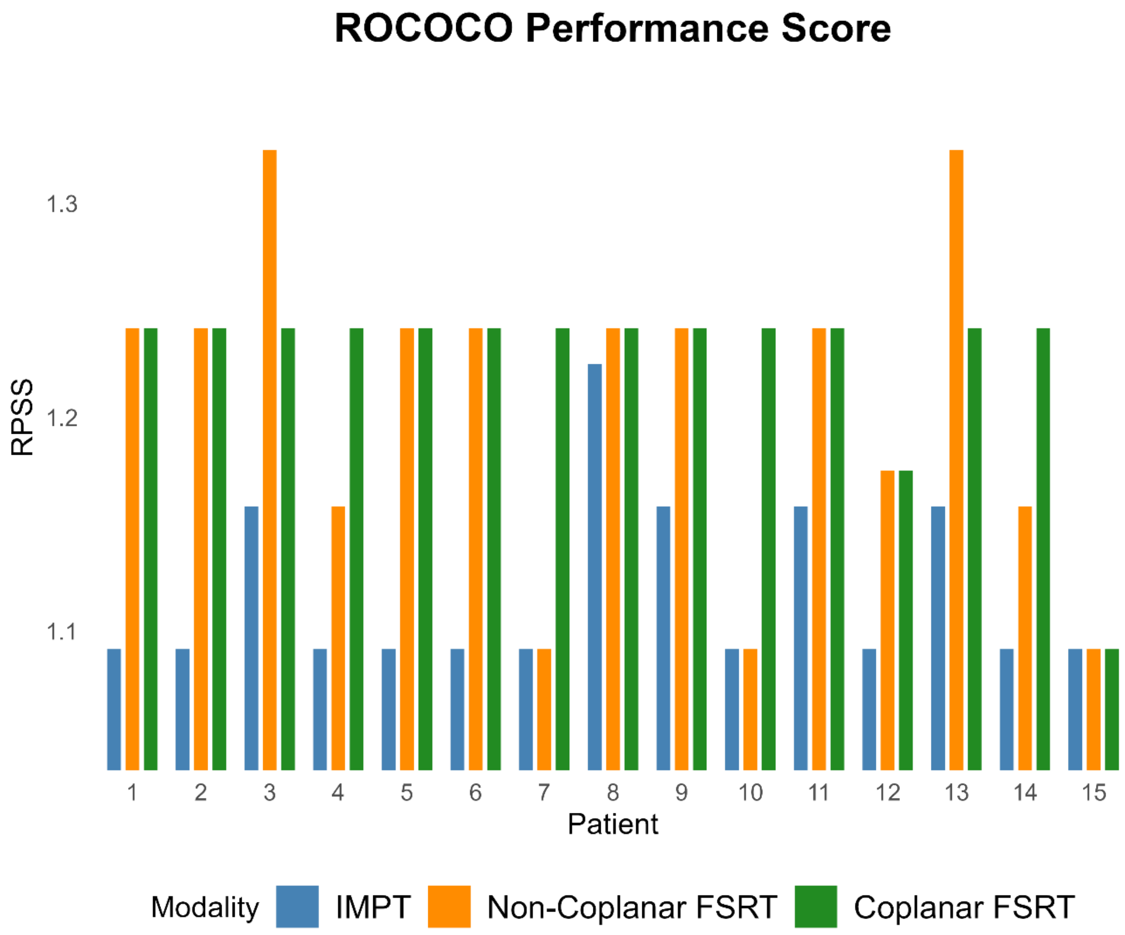

Figure S1. Individual ROCOCO Performance Scores (RPSS) by Patient and Planning Technique; Intensity-Modulated Proton Therapy (IMPT), Non-Coplanar Fractionated Stereotactic Radiotherapy (FSRT), and Coplanar FSRT. The RPSS is a unitless composite score used to estimate radiotherapy-induced adverse events (AEs); a higher score indicates an increase risk for radiotherapy-induced AEs.

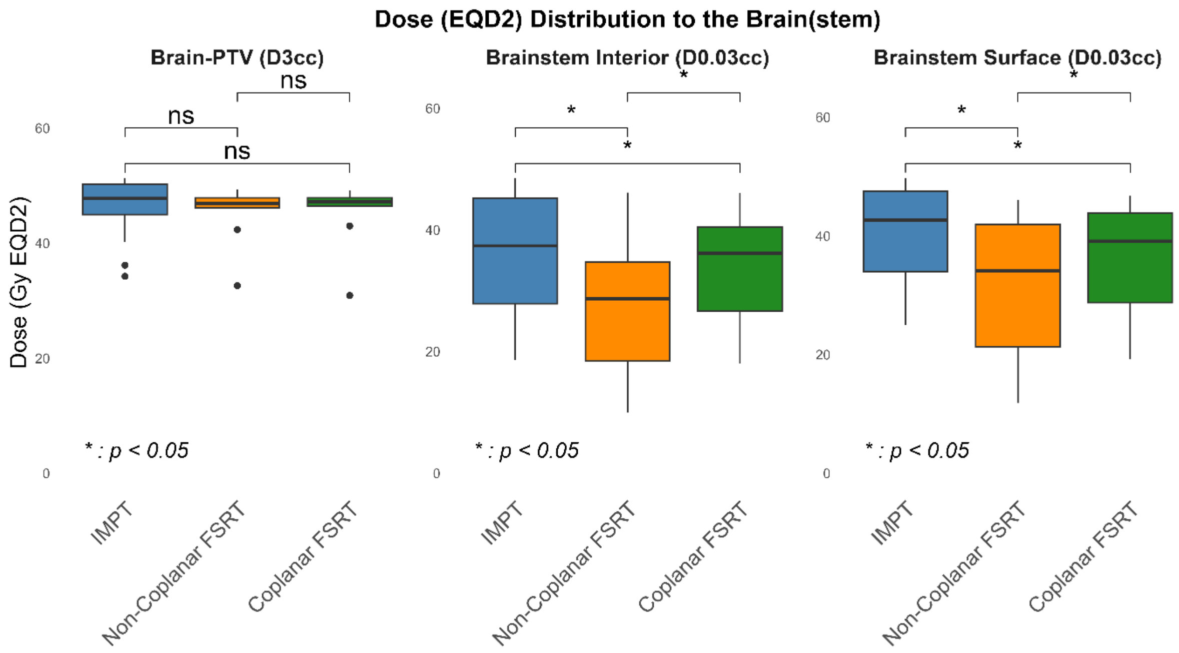

Figure S2. Dose (Gy EQD_2) distribution for the Brain and Brainstem. Values are presented as Median (IQR: Q_1-Q_3). Outliers are highlighted as black dots. Brain-PTV (D_{3cc}): IMPT: 47.78 (44.99–50.27); Coplanar FSRT: 47.19 (46.5–47.9); Non-Coplanar FSRT: 46.89 (46.1–47.9). Brainstem Interior ($D_{0.03cc}$): IMPT: 37.38 (27.9–45.2); Coplanar FSRT: 36.17 (26.6–40.5); Non-Coplanar FSRT: 28.70 (18.4–34.7). Brainstem Surface ($D_{0.03cc}$): IMPT: 42.65 (33.9–47.6); Coplanar FSRT: 39.08 (28.8–43.8); Non-Coplanar FSRT: 34.09 (21.6–41.9).

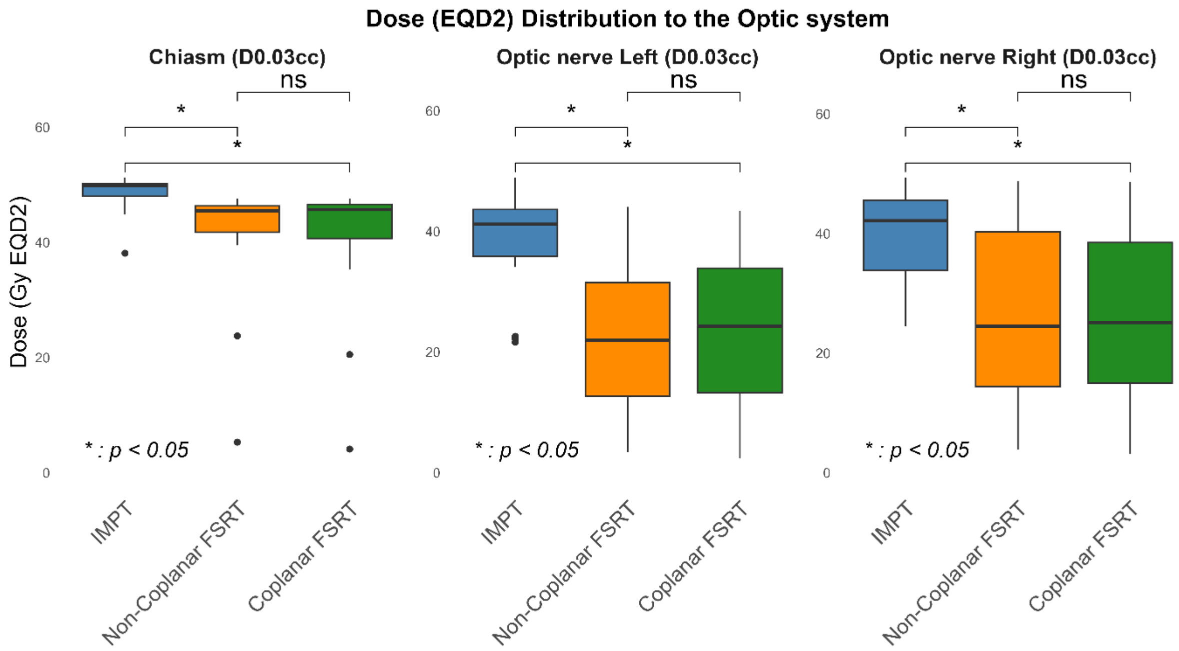

Figure S3. Dose (Gy EQD_2) distribution for the optic pathway. Values are presented as Median (IQR: Q_1-Q_3). Outliers are highlighted as black dots. Chiasm ($D_{0.03cc}$): IMPT: 49.88 (48.0–50.2); Coplanar FSRT: 45.71 (40.7–46.6); Non-Coplanar FSRT: 45.47 (41.8–46.3). Optic nerve Left ($D_{0.03cc}$): IMPT: 41.21 (35.9–43.7); Coplanar FSRT: 24.30 (13.3–33.9); Non-Coplanar FSRT: 22.00 (12.7–31.6). Optic nerve Right ($D_{0.03cc}$): IMPT: 42.14 (33.8–45.6); Coplanar FSRT: 25.13 (15.0–38.5); Non-Coplanar FSRT: 24.49 (14.4–40.3).

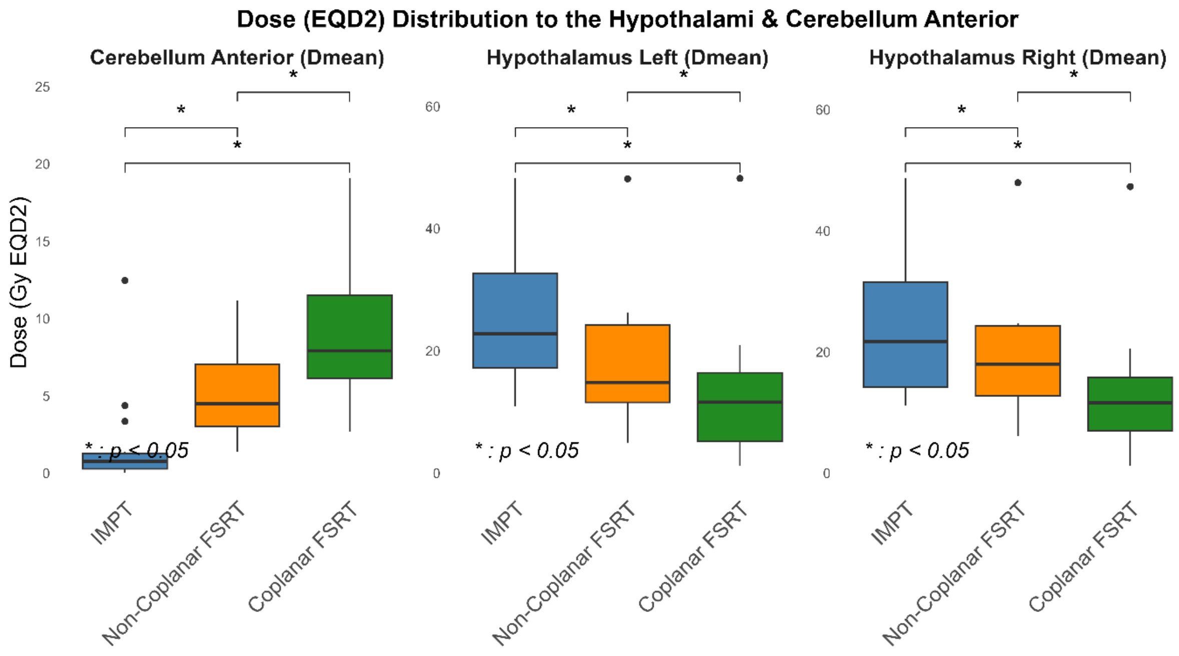

Figure S4. Mean dose distribution for the hypothalami and the cerebellum anterior. Values are presented as Median (IQR: Q_1-Q_3). Outliers are highlighted as black dots. Hypothalamus Left: IMPT: 22.79 (17.3–32.6); Coplanar FSRT: 11.61 (5.2–16.4); Non-Coplanar FSRT: 14.86 (11.6–24.2). Hypothalamus Right: IMPT: 21.76 (14.2–31.6); Coplanar FSRT: 11.64 (6.9–15.8); Non-Coplanar FSRT: 18.02 (12.8–24.3). Anterior cerebellum: IMPT: 0.77 (0.3-1.3), Coplanar FSRT 7.92 (6.1-11.6), Non-Coplanar FSRT: 4.50 (3.0-7.1).

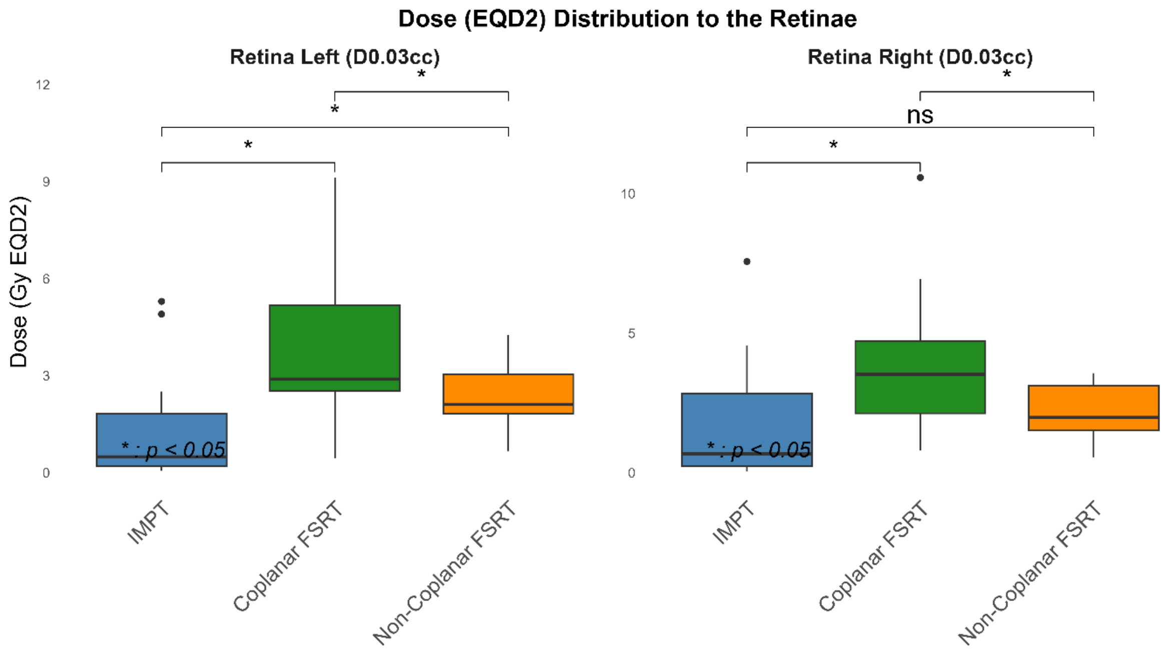

Figure S5. Mean dose distribution for the retinae. Values are presented as Median (IQR: Q_1-Q_3). Outliers are highlighted as black dots. Retina Left: IMPT: 0.49 (0.2–1.8); Coplanar FSRT: 2.90 (2.5–5.2); Non-Coplanar FSRT: 2.11 (1.8–3.1). Retina Right: IMPT: 0.68 (0.2–2.8); Coplanar FSRT: 3.53 (2.1–4.7); Non-Coplanar FSRT: 1.98 (1.5–3.1).

Table S1. Post-hoc Wilcoxon Signed-Rank Test Results of individual testing of dose to Ogans of Interest (OOIs) not represented by the RPSS

OOI	Comparison	Result
Chiasm	IMPT vs Coplanar	p = 0.00018, *
Chiasm	IMPT vs non-coplanar	p = 0.00018, *
Chiasm	Non-coplanar vs Coplanar	p = 0.19, ns
Optic nerve left	IMPT vs Coplanar	p = 0.00018, *
Optic nerve left	IMPT vs non-coplanar	p = 0.00018, *
Optic nerve left	Non-coplanar vs Coplanar	p = 1.00000, ns
Optic nerve right	IMPT vs Coplanar	p = 0.00037, *
Optic nerve right	IMPT vs non-coplanar	p = 0.00037, *
Optic nerve right	Non-coplanar vs Coplanar	p = 1.00000, ns
Brainstem surface	IMPT vs Coplanar	p = 0.00018, *
Brainstem surface	IMPT vs non-coplanar	p = 0.00018, *
Brainstem surface	Non-coplanar vs Coplanar	p = 0.00018, *
Brainstem interior	IMPT vs Coplanar	p = 0.12, ns
Brainstem interior	IMPT vs non-coplanar	p = 0.00018, *
Brainstem interior	Non-coplanar vs Coplanar	p = 0.00037, *
VSCC left	IMPT vs Coplanar	p = 0.00018, *
VSCC left	IMPT vs non-coplanar	p = 0.00018, *
VSCC left	Non-coplanar vs Coplanar	p =0.013, *
VSCC right	IMPT vs Coplanar	p = 0.006, *
VSCC right	IMPT vs non-coplanar	p = 0.013, *
VSCC right	Non-coplanar vs Coplanar	p = 0.19, ns
Hypothalamus left	IMPT vs Coplanar	p = 0.003, *
Hypothalamus left	IMPT vs non-coplanar	p = 0.045, *
Hypothalamus left	Non-Coplanar vs Coplanar	p = 0.0009, *
Hypothalamus right	IMPT vs Coplanar	p = 0.0055, *
Hypothalamus right	IMPT vs non-coplanar	p = 0.002, *
Hypothalamus right	Non-coplanar vs Coplanar	p = 0.003, *
Retina left	IMPT vs Coplanar	p = 0.0046, *

OOI	Comparison	Result
Retina left	IMPT vs non-coplanar	p = 0.09, ns
Retina left	Non-coplanar vs coplanar	p = 0.01, *
Retina right	IMPT vs Coplanar	p = 0.065, ns
Retina right	IMPT vs non-coplanar	p = 0.51, ns
Retina right	Non-coplanar vs coplanar	p = 0.00018, *
Cerebellum anterior	IMPT vs Coplanar	p = 0.0013, *
Cerebellum anterior	IMPT vs non-coplanar	p = 0.016, *
Cerebellum anterior	IMPT vs non-coplanar	p = 0.0026, *

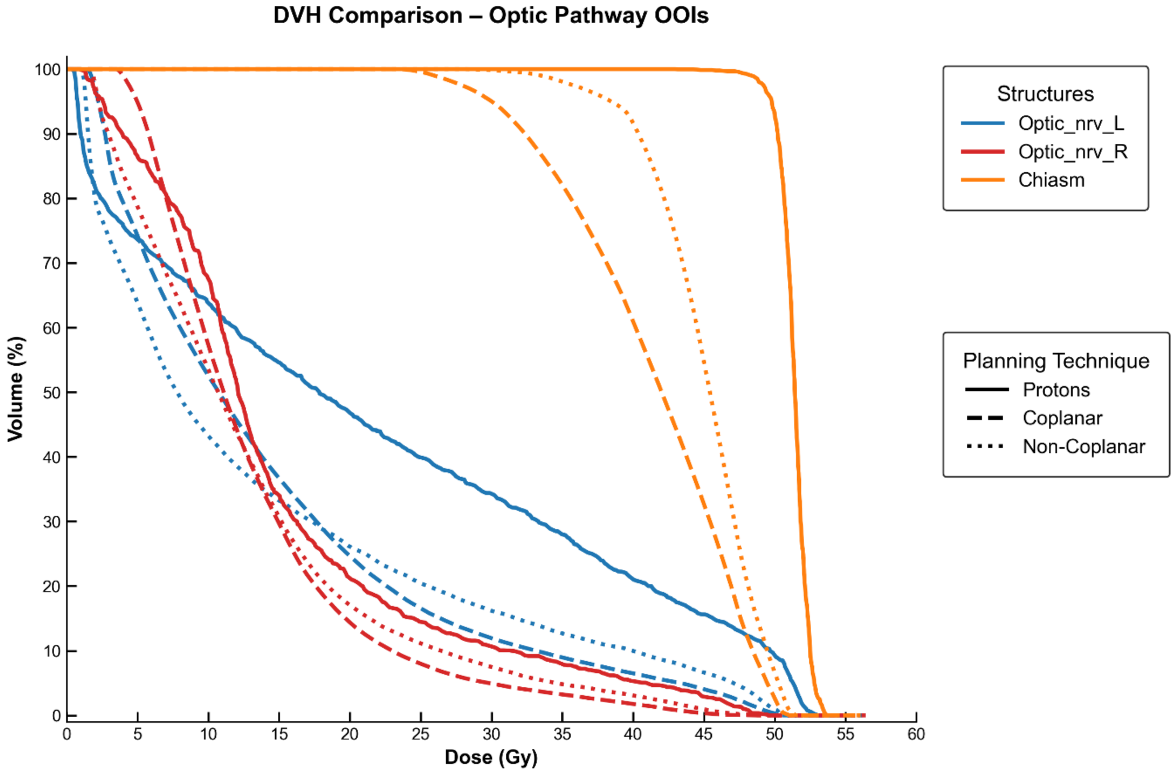

Figure S6. Dose-Volume Histogram (DVH) comparison of the optic pathway OOIs. Cumulative DVH for the Optic Chiasm (orange), Left Optic Nerve (blue), and Right Optic Nerve (red) across three planning techniques.

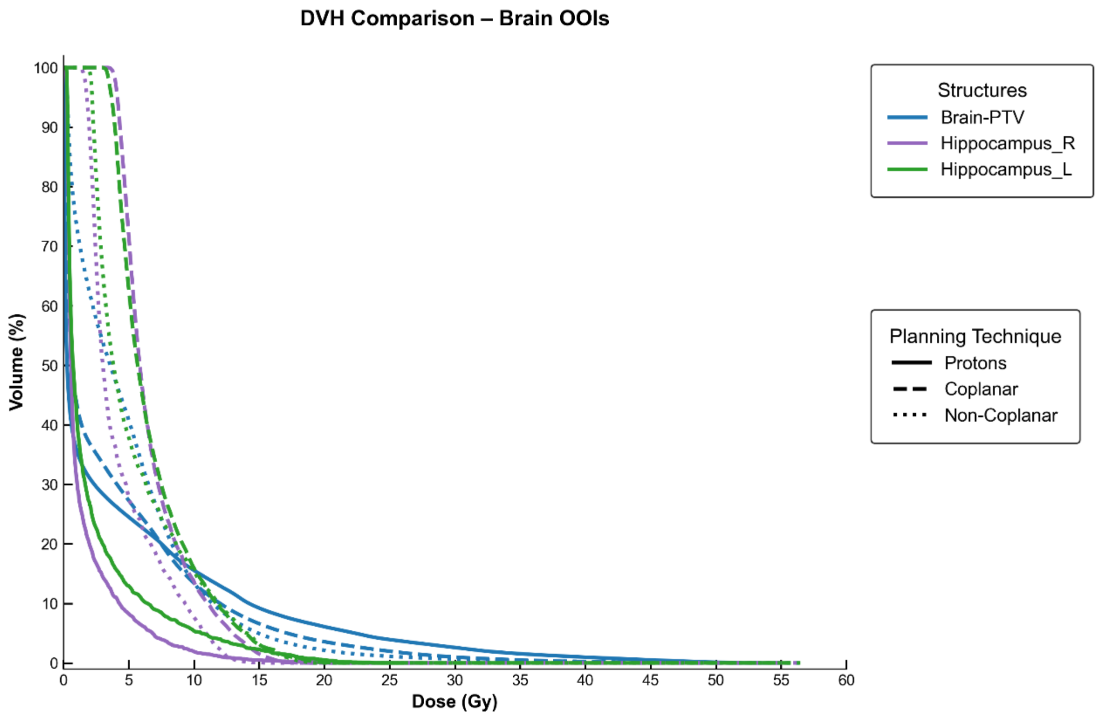

Figure S7. Dose-Volume Histogram (DVH) comparison of the Brain OOIs. Cumulative DVH for the Left Hippocampus (green), Right Hippocampus (magenta), and Brain-PTV (blue) across three planning techniques.

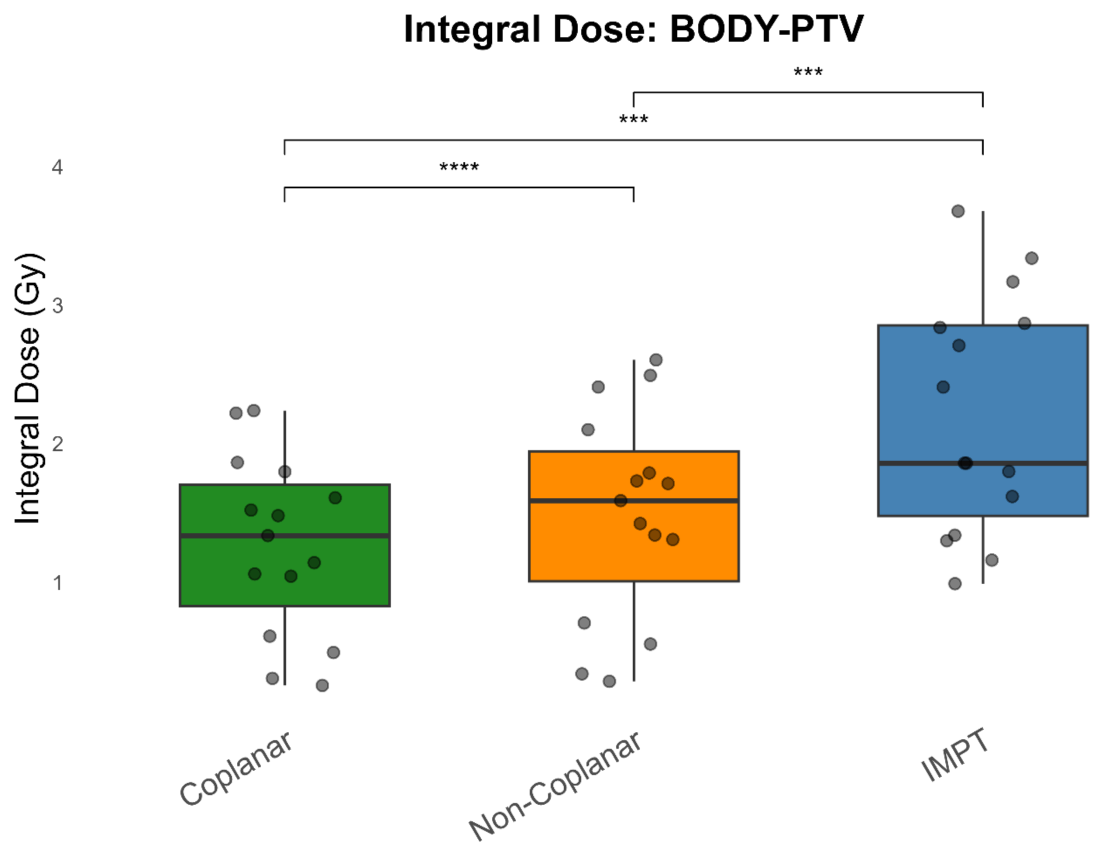

Figure S8. Integral dose comparison for the patient body (Body-PTV) across three techniques. Individual patient data points are overlaid as grey jittered dots to demonstrate inter-patient variability. Median (IQR: Q_1-Q_3) results were: Coplanar FSRT, 1.34 Gy (0.8–1.7 Gy); Non-Coplanar FSRT, 1.59 Gy (1.0–1.9 Gy); and IMPT, 1.86 Gy (1.5–2.9 Gy). Statistical significance was determined via paired Wilcoxon signed-rank tests with Bonferroni correction: *** denotes $p < 0.001$, **** denotes $p < 0.0001$.
